# 2-Ethyl-2,3-dihydro-1,2-benzothia­zole-1,1,3-trione

**DOI:** 10.1107/S1600536811009184

**Published:** 2011-03-15

**Authors:** Muneeb Hayat Khan, Islam Ullah Khan, Muhammad Nadeem Arshad, Mehmet Akkurt

**Affiliations:** a Materials Chemistry Laboratory, Department of Chemistry, GC University, Lahore 54000, Pakistan; b Department of Physics, Faculty of Sciences, Erciyes University, 38039 Kayseri, Turkey

## Abstract

In the title mol­ecule, C_9_H_9_NO_3_S, the bond lengths and angles fall within normal ranges. All nine ring atoms almost lie in a common plane (r.m.s. deviation 0.021 Å). In the crystal, symmetry-related mol­ecules are linked *via* C—H⋯O hydrogen bonds, forming a three-dimensional network.

## Related literature

For related literature on benzisothia­zolone-1,1-dioxide derivatives, see: Hu *et al.* (2004 ▶); Kap-Sun & Nicholas (1998 ▶); Liang *et al.* (2006 ▶); Masashi *et al.* (1999 ▶); Nagasawa *et al.* (1995 ▶). For related structures, see: Hu *et al.* (2006 ▶); Xu *et al.* (2005 ▶); Wen *et al.* (2006 ▶).
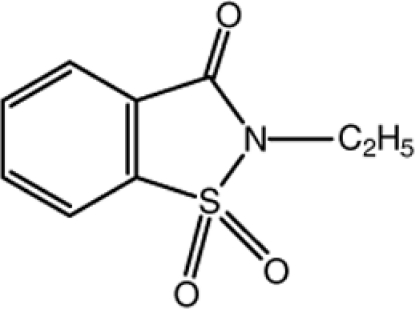


## Experimental

### 

#### Crystal data

C_9_H_9_NO_3_S
*M* *_r_* = 211.24
Monoclinic, 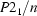

*a* = 10.4559 (5) Å
*b* = 7.5484 (5) Å
*c* = 12.9408 (7) Å
β = 105.863 (2)°
*V* = 982.46 (10) Å^3^
*Z* = 4
Mo *K*α radiation
μ = 0.31 mm^−1^
*T* = 296 K
0.19 × 0.18 × 0.09 mm

#### Data collection

Bruker APEXII CCD diffractometer
9319 measured reflections
2429 independent reflections
1822 reflections with *I* > 2σ(*I*)
*R*_int_ = 0.081

#### Refinement

*R*[*F* ^2^ > 2σ(*F* ^2^)] = 0.046
*wR*(*F* ^2^) = 0.144
*S* = 1.08
2429 reflections
129 parameters
H-atom parameters constrained
Δρ_max_ = 0.34 e Å^−3^
Δρ_min_ = −0.35 e Å^−3^

### 

Data collection: *APEX2* (Bruker, 2007 ▶); cell refinement: *SAINT* (Bruker, 2007 ▶); data reduction: *SAINT*; program(s) used to solve structure: *SHELXS97* (Sheldrick, 2008 ▶); program(s) used to refine structure: *SHELXL97* (Sheldrick, 2008 ▶); molecular graphics: *ORTEP-3 for Windows* (Farrugia, 1997 ▶); software used to prepare material for publication: *WinGX* (Farrugia, 1999 ▶) and *PLATON* (Spek, 2009 ▶).

## Supplementary Material

Crystal structure: contains datablocks global, I. DOI: 10.1107/S1600536811009184/bt5490sup1.cif

Structure factors: contains datablocks I. DOI: 10.1107/S1600536811009184/bt5490Isup2.hkl

Additional supplementary materials:  crystallographic information; 3D view; checkCIF report

## Figures and Tables

**Table 1:** Hydrogen-bond geometry (Å, °)

*D*—H⋯*A*	*D*—H	H⋯*A*	*D*⋯*A*	*D*—H⋯*A*
C2—H2⋯O1^i^	0.93	2.37	3.265 (2)	162
C3—H3⋯O2^ii^	0.93	2.53	3.295 (3)	140
C8—H8*A*⋯O3^iii^	0.97	2.45	3.139 (3)	128

Symmetry codes: (i) 
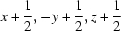
; (ii) 
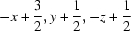
; (iii) 
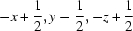
.

**Fig. 1.**
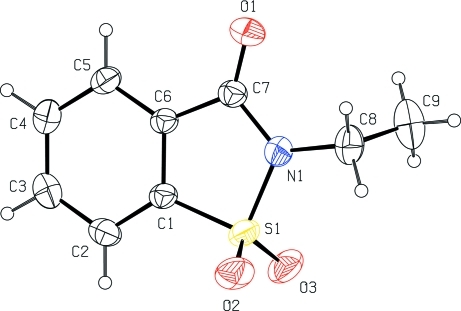
View of the title molecule with atom numbering scheme. Displacement ellipsoids for non-H atoms are drawn at the 30% probability level.

**Fig. 2.**
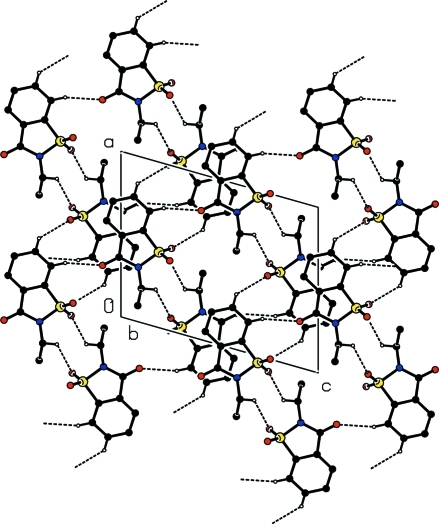
A view of the packing and hydrogen bonding interactions of the title compound down the b axis. Hydrogen atoms not involved in hydrogen bonding have been omitted for clarity.

## supplementary crystallographic information

### Comment

Benzisothiazolone-1,1-dioxide is part of a class of heterocycles which has been investigated in pharmaceutical research (Kap-Sun & Nicholas, 1998). 1,2-Benzisothiazole-3-one 1,1-dioxide (saccharin) has been widely incorporated into a variety of biologically active compounds. It has been identified as an important molecular component in various classes of 5-Htla antagonists, analgesics and human mast cell tryptase inhibitors (Liang *et al.*, 2006). In particular, N-substituted derivatives, e.g with *N*-hydroxy and *N*-alkyl substituents, have shown important biological activity (Nagasawa *et al.*, 1995). Among *N*-alkyl derivatives, various synthetic routes have been reported for the synthesis of the title compound involving ionic liquids and free radical mechanisms (Hu *et al.*, 2004; Masashi *et al.*, 1999).

In the molecule of the title compound (Fig. 1), all the bond lengths and bond angles agree with the corresponding values in similar structures containing benzisothiazole group (Hu *et al.*, 2006; Xu *et al.*, 2005; Wen *et al.*, 2006).

Atoms C1–C7, S1, O1 and N1 of the title molecule are essentially coplanar, with a maximum deviation of 0.020 (1) Å for S1. The packing  of the molecules is stabilized by intermolecular C—H···O intermolecular hydrogen bonds (Table 1, Fig. 2).

### Experimental

Sodium sacharrin (0.5 g, 2.439 mmol) was taken in round bottom flask and 20 ml DMF was added to it. It was kept for stirring at room temperature for 5 minutes then ethyl iodide (0.195 ml, 2.439 mmol) was added to the solution. Then reaction mixture was kept under reflux for 3 h at 333 K and after 3 h the TLC confirmed the completion of reaction. The product was obtained in ice-water, filtered and dried. Dried precipitates were dissolved in methanol for crystallization (yield: 93%).

### Refinement

In the last cycles of the refinement, 3 reflections (1 0 1), (-1 0 1) and (-2 2 2) were eliminated due to being poorly measured in the vicinity of the beam stop. All H atoms were positioned geometrically with C—H = 0.93 - 0.97 Å, and allowed to ride on their parent atoms, with *U*_iso_(H) = 1.2*U*_eq_(C) for aromatic and methylene, and *U*_iso_(H) = 1.5*U*_eq_(C) for methyl H atoms.

### Crystal data

**Table:**

C_9_H_9_NO_3_S	*F*(000) = 440
*M**_r_* = 211.24	*D*_x_ = 1.428 Mg m^−^^3^
Monoclinic, *P*2_1_/*n*	Mo *K*α radiation, λ = 0.71073 Å
Hall symbol: -P 2yn	Cell parameters from 3812 reflections
*a* = 10.4559 (5) Å	θ = 2.7–28.3°
*b* = 7.5484 (5) Å	µ = 0.31 mm^−^^1^
*c* = 12.9408 (7) Å	*T* = 296 K
β = 105.863 (2)°	Plate, colourless
*V* = 982.46 (10) Å^3^	0.19 × 0.18 × 0.09 mm
*Z* = 4	

### Data collection

**Table:**

Bruker APEXII CCD diffractometer	1822 reflections with *I* > 2σ(*I*)
Radiation source: sealed tube	*R*_int_ = 0.081
graphite	θ_max_ = 28.3°, θ_min_ = 3.2°
φ and ω scans	*h* = −13→13
9319 measured reflections	*k* = −10→10
2429 independent reflections	*l* = −17→17

### Refinement

**Table:**

Refinement on *F*^2^	Primary atom site location: structure-invariant direct methods
Least-squares matrix: full	Secondary atom site location: difference Fourier map
*R*[*F*^2^ > 2σ(*F*^2^)] = 0.046	Hydrogen site location: inferred from neighbouring sites
*wR*(*F*^2^) = 0.144	H-atom parameters constrained
*S* = 1.08	*w* = 1/[σ^2^(*F*_o_^2^) + (0.0789*P*)^2^ + 0.0563*P*] where *P* = (*F*_o_^2^ + 2*F*_c_^2^)/3
2429 reflections	(Δ/σ)_max_ < 0.001
129 parameters	Δρ_max_ = 0.34 e Å^−^^3^
0 restraints	Δρ_min_ = −0.35 e Å^−^^3^

### Special details

**Table:**

Geometry. Bond distances, angles *etc*. have been calculated using the rounded fractional coordinates. All su's are estimated from the variances of the (full) variance-covariance matrix. The cell e.s.d.'s are taken into account in the estimation of distances, angles and torsion angles
Refinement. Refinement on *F*^2^ for ALL reflections except those flagged by the user for potential systematic errors. Weighted *R*-factors *wR* and all goodnesses of fit *S* are based on *F*^2^, conventional *R*-factors *R* are based on *F*, with *F* set to zero for negative *F*^2^. The observed criterion of *F*^2^ > σ(*F*^2^) is used only for calculating -*R*-factor-obs *etc*. and is not relevant to the choice of reflections for refinement. *R*-factors based on *F*^2^ are statistically about twice as large as those based on *F*, and *R*-factors based on ALL data will be even larger.

### Fractional atomic coordinates and isotropic or equivalent isotropic displacement parameters (Å^2^)

**Table:**

	*x*	*y*	*z*	*U*_iso_*/*U*_eq_	
S1	0.45672 (5)	0.21780 (7)	0.19351 (3)	0.0544 (2)	
O1	0.27985 (14)	0.1334 (2)	−0.09326 (10)	0.0684 (5)	
O2	0.50046 (15)	0.0632 (2)	0.25648 (10)	0.0770 (6)	
O3	0.42257 (17)	0.3675 (2)	0.24709 (11)	0.0771 (6)	
N1	0.33045 (15)	0.1647 (2)	0.08810 (12)	0.0540 (5)	
C1	0.55956 (17)	0.2745 (2)	0.11296 (13)	0.0442 (5)	
C2	0.68739 (19)	0.3429 (3)	0.14490 (15)	0.0568 (6)	
C3	0.74791 (19)	0.3782 (3)	0.06576 (17)	0.0620 (6)	
C4	0.68513 (19)	0.3479 (3)	−0.04052 (16)	0.0591 (7)	
C5	0.55651 (19)	0.2815 (2)	−0.07236 (14)	0.0515 (6)	
C6	0.49424 (17)	0.2447 (2)	0.00599 (13)	0.0417 (5)	
C7	0.35743 (17)	0.1749 (2)	−0.00979 (13)	0.0473 (5)	
C8	0.2068 (2)	0.0906 (3)	0.10313 (19)	0.0704 (8)	
C9	0.0967 (3)	0.2234 (3)	0.0851 (3)	0.0977 (13)	
H2	0.73010	0.36390	0.21690	0.0680*	
H3	0.83380	0.42380	0.08450	0.0740*	
H4	0.72950	0.37220	−0.09210	0.0710*	
H5	0.51370	0.26240	−0.14450	0.0620*	
H8A	0.22420	0.04450	0.17560	0.0840*	
H8B	0.17840	−0.00750	0.05380	0.0840*	
H9A	0.12320	0.31940	0.13500	0.1470*	
H9B	0.01870	0.16790	0.09560	0.1470*	
H9C	0.07780	0.26810	0.01300	0.1470*	

### Atomic displacement parameters (Å^2^)

**Table:**

	*U*^11^	*U*^22^	*U*^33^	*U*^12^	*U*^13^	*U*^23^
S1	0.0641 (3)	0.0629 (4)	0.0381 (3)	0.0102 (2)	0.0174 (2)	0.0029 (2)
O1	0.0600 (8)	0.0833 (10)	0.0532 (8)	−0.0087 (7)	0.0010 (6)	−0.0090 (7)
O2	0.0932 (11)	0.0822 (10)	0.0543 (8)	0.0116 (9)	0.0179 (7)	0.0255 (7)
O3	0.0937 (11)	0.0869 (10)	0.0586 (8)	0.0168 (9)	0.0343 (8)	−0.0156 (8)
N1	0.0505 (8)	0.0625 (9)	0.0515 (8)	0.0006 (7)	0.0181 (7)	0.0040 (7)
C1	0.0481 (9)	0.0458 (9)	0.0371 (8)	0.0107 (7)	0.0092 (7)	−0.0022 (6)
C2	0.0524 (10)	0.0596 (11)	0.0503 (10)	0.0075 (8)	0.0001 (8)	−0.0070 (8)
C3	0.0456 (9)	0.0604 (11)	0.0789 (13)	0.0027 (8)	0.0150 (9)	−0.0019 (10)
C4	0.0577 (11)	0.0601 (11)	0.0665 (12)	0.0051 (9)	0.0287 (9)	0.0037 (9)
C5	0.0611 (11)	0.0547 (10)	0.0406 (8)	0.0067 (8)	0.0172 (8)	−0.0013 (7)
C6	0.0464 (8)	0.0406 (8)	0.0372 (8)	0.0068 (7)	0.0098 (6)	−0.0015 (6)
C7	0.0484 (9)	0.0475 (9)	0.0439 (9)	0.0047 (7)	0.0092 (7)	−0.0008 (7)
C8	0.0610 (12)	0.0704 (14)	0.0886 (14)	−0.0035 (10)	0.0356 (11)	0.0074 (11)
C9	0.0638 (14)	0.0892 (18)	0.149 (3)	0.0057 (13)	0.0440 (17)	0.0024 (17)

### Geometric parameters (Å, °)

**Table:**

S1—O2	1.4252 (15)	C5—C6	1.375 (3)
S1—O3	1.4214 (16)	C6—C7	1.485 (3)
S1—N1	1.6673 (16)	C8—C9	1.496 (4)
S1—C1	1.7432 (18)	C2—H2	0.9300
O1—C7	1.202 (2)	C3—H3	0.9300
N1—C7	1.373 (2)	C4—H4	0.9300
N1—C8	1.470 (3)	C5—H5	0.9300
C1—C2	1.386 (3)	C8—H8A	0.9700
C1—C6	1.384 (2)	C8—H8B	0.9700
C2—C3	1.369 (3)	C9—H9A	0.9600
C3—C4	1.372 (3)	C9—H9B	0.9600
C4—C5	1.388 (3)	C9—H9C	0.9600
			
O2—S1—O3	117.14 (8)	N1—C7—C6	109.03 (14)
O2—S1—N1	109.24 (8)	N1—C8—C9	113.06 (19)
O2—S1—C1	112.89 (9)	C1—C2—H2	121.00
O3—S1—N1	109.99 (9)	C3—C2—H2	122.00
O3—S1—C1	112.01 (9)	C2—C3—H3	119.00
N1—S1—C1	92.85 (8)	C4—C3—H3	119.00
S1—N1—C7	115.14 (13)	C3—C4—H4	119.00
S1—N1—C8	120.78 (13)	C5—C4—H4	119.00
C7—N1—C8	123.60 (16)	C4—C5—H5	121.00
S1—C1—C2	127.97 (13)	C6—C5—H5	121.00
S1—C1—C6	109.98 (13)	N1—C8—H8A	109.00
C2—C1—C6	122.05 (16)	N1—C8—H8B	109.00
C1—C2—C3	117.03 (17)	C9—C8—H8A	109.00
C2—C3—C4	121.7 (2)	C9—C8—H8B	109.00
C3—C4—C5	121.14 (19)	H8A—C8—H8B	108.00
C4—C5—C6	118.02 (17)	C8—C9—H9A	109.00
C1—C6—C5	120.07 (17)	C8—C9—H9B	109.00
C1—C6—C7	112.86 (15)	C8—C9—H9C	109.00
C5—C6—C7	127.07 (15)	H9A—C9—H9B	110.00
O1—C7—N1	123.80 (17)	H9A—C9—H9C	109.00
O1—C7—C6	127.17 (16)	H9B—C9—H9C	110.00
			
O2—S1—N1—C7	−111.89 (13)	C7—N1—C8—C9	−85.9 (3)
O2—S1—N1—C8	60.49 (17)	C2—C1—C6—C7	−178.70 (16)
O3—S1—N1—C7	118.24 (13)	S1—C1—C6—C5	179.56 (13)
O3—S1—N1—C8	−69.38 (17)	S1—C1—C2—C3	−179.53 (16)
C1—S1—N1—C7	3.57 (13)	C6—C1—C2—C3	−0.8 (3)
C1—S1—N1—C8	175.95 (15)	S1—C1—C6—C7	0.24 (17)
O2—S1—C1—C2	−70.93 (19)	C2—C1—C6—C5	0.6 (3)
O2—S1—C1—C6	110.22 (13)	C1—C2—C3—C4	0.2 (3)
O3—S1—C1—C2	63.88 (19)	C2—C3—C4—C5	0.7 (3)
O3—S1—C1—C6	−114.97 (13)	C3—C4—C5—C6	−0.8 (3)
N1—S1—C1—C2	176.79 (18)	C4—C5—C6—C7	179.41 (17)
N1—S1—C1—C6	−2.06 (13)	C4—C5—C6—C1	0.2 (2)
S1—N1—C7—O1	176.40 (14)	C5—C6—C7—N1	−177.04 (16)
C8—N1—C7—O1	4.3 (3)	C1—C6—C7—O1	−178.07 (17)
S1—N1—C7—C6	−3.89 (17)	C1—C6—C7—N1	2.23 (19)
C8—N1—C7—C6	−176.03 (16)	C5—C6—C7—O1	2.7 (3)
S1—N1—C8—C9	102.4 (2)		

### Hydrogen-bond geometry (Å, °)

**Table:**

*D*—H···*A*	*D*—H	H···*A*	*D*···*A*	*D*—H···*A*
C2—H2···O1^i^	0.93	2.37	3.265 (2)	162
C3—H3···O2^ii^	0.93	2.53	3.295 (3)	140
C8—H8A···O3^iii^	0.97	2.45	3.139 (3)	128

Symmetry codes: (i) *x*+1/2, −*y*+1/2, *z*+1/2; (ii) −*x*+3/2, *y*+1/2, −*z*+1/2; (iii) −*x*+1/2, *y*−1/2, −*z*+1/2.
